# The risk of acute liver injury among users of antibiotic medications: a comparison of case‐only studies.

**DOI:** 10.1002/pds.3846

**Published:** 2015-08-06

**Authors:** Ruth Brauer, Ana Ruigómez, Olaf Klungel, Robert Reynolds, Maurille Feudjo Tepie, Liam Smeeth, Ian Douglas

**Affiliations:** ^1^ Faculty of Epidemiology and Population HealthLondon School of Hygiene and Tropical MedicineLondonUnited Kingdom; ^2^ Fundación Centro Español de Investigación Farmacoepidemiológica, (CEIFE)MadridSpain; ^3^ Faculty of Science, Division of Pharmacoepidemiology and Clinical PharmacologyUtrecht UniversityUtrechtThe Netherlands; ^4^ EpidemiologyPfizer Research and DevelopmentNew YorkUSA; ^5^ Amgen NVLondonUnited Kingdom

**Keywords:** self‐controlled case series, case‐crossover, acute liver injury, antibiotic agents, CPRD, pharmacoepidemiology

## Abstract

**Purpose:**

The aims of this study were two‐fold: (i) to investigate the effect of exposure to antibiotic agents on the risk of acute liver injury using a self‐controlled case series and case‐crossover study and (ii) to compare the results between the case‐only studies.

**Methods:**

For the self‐controlled case series study relative incidence ratios (IRR) were calculated by dividing the rate of acute liver injury experienced during patients' periods of exposure to antibiotics to patients' rate of events during non‐exposed time using conditional Poisson regression. For the case‐crossover analysis we calculated Odds Ratios (OR) using conditional logistic regression by comparing exposure during 14‐ and 30‐day risk windows with exposure during control moments.

**Results:**

Using the self‐controlled case series approach, the IRR was highest during the first 7 days after receipt of a prescription (10.01, 95% CI 6.59–15.18). Omitting post‐exposure washout periods lowered the IRR to 7.2. The highest estimate in the case‐crossover analysis was found when two 30‐day control periods 1 year prior to the 30‐day ALI risk period were retained in the analysis: OR = 6.5 (95% CI, 3.95–10.71). The lowest estimate was found when exposure in the 14‐day risk period was compared to exposure in four consecutive 14‐day control periods immediately prior to the risk period (OR = 3.05, 95% CI, 2.06–4.53).

**Conclusion:**

An increased relative risk of acute liver injury was consistently observed using both self‐controlled case series and case‐crossover designs. Case‐only designs can be used as a viable alternative study design to study the risk of acute liver injury, albeit with some limitations. © 2015 The Authors *Pharmacoepidemiology and Drug Safety* Published by John Wiley & Sons Ltd.

## Introduction

Electronic health records offer great opportunities for pharmacoepidemiological research into rare diseases such as acute liver injury. One of the current key challenges is how to correct for confounding factors that are difficult to measure using these electronic health records, such as genetic susceptibility. Case‐only designs, in which cases act as their own control, can provide some advantages over the more traditional designs as they implicitly control for confounders that do not vary over time.^1^, ^2^, ^3^ As acute liver injury (ALI) is often idiosyncratic and the underlying risk of patients who experience ALI may differ from patients who never experience liver injury, the self‐controlled case series and the case‐crossover design were used to investigate the relationship between antibiotic agents and ALI.

The aims of this study were two‐fold: (i) to investigate the effect of exposure to antibiotic agents on the risk of acute liver injury using a self‐controlled case series and case‐crossover study and (ii) to compare the results between the case‐only studies; and explore potential biases likely to result from the study designs. The latter objective addresses one of the main purposes of the IMI‐PROTECT project, in which this study was embedded.^4^

## Methods

### Source‐ and study population

The database and the source population were identical to those described in a previous paper by our group.^5^ Briefly, follow‐up started at the earliest of 12 months after the start of Clinical Practice Research Datalink (CPRD) registration from 2004 onwards and ended at the earliest of the end of the CPRD record and December 2009. All patients from the source population who received a prescription for an antibiotic agent and who were diagnosed with acute liver injury during their follow‐up time were included in the self‐controlled case series and case‐crossover studies. The start date for all cases was 1 January 2004 or after 1 year of research quality follow‐up (whichever came later). In the case‐crossover analysis the follow‐up period ended at the date of acute liver injury.

Patients diagnosed with alcohol‐related problems, cancer, gallbladder disease, pancreatic disease, viral hepatitis and other (chronic) liver diseases prior to or on the date of their liver injury were excluded. The algorithm that was used to define acute liver injury has been described in detail by Ruigomez *et al.* 5 Briefly, medical files were searched for specific codes related to liver disease or symptoms, followed by an assessment of laboratory test results. In addition, cases had to be referred to a specialist or hospital related to liver disease within 2 weeks of a recorded diagnosis of liver injury.

Exposure was defined as a recorded prescription for an antibiotic agent listed in chapter 5 of the British National Formulary (BNF). Prescriptions for topical antibiotics were excluded. Duration of exposure was calculated by dividing the total quantity of prescribed antibiotics by the numeric daily dosage prescribed. The median duration of exposure to all antibiotic agents was imputed when information on the total quantity or the prescribed daily dosage was missing (2%). An individual was assumed to be exposed between the end of their current prescription and a repeat prescription if less than 14 days had passed.

### Self‐controlled case series design

**Figure 1:**
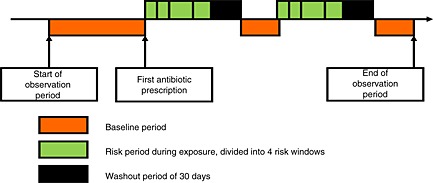
Diagram self controlled case series method ‘antibiotics and ALI’

The rate ratio of the self‐controlled case series (SCCS) method is based on within‐person comparisons rather than between‐person comparisons.^6^ Patients who received an antibiotic agent during follow‐up and experienced an acute liver injury during the study period were included and contributed both exposed and unexposed person time. Each individual's exposed observation time was divided into risk windows as follows: (i) from 0 to 7 days after the start of the treatment, (ii) from 8 to 14 days after the start of the treatment and (iii) from 15 to 30 days after the start of treatment and (iv) the remaining exposed time, followed by (v) a period of 30 days to account for a gradual shift from exposed to unexposed time (a ‘wash‐out’ period). The remaining person‐time was used as a baseline comparison period. The 30‐day washout period was divided into three 10‐day periods after treatment (see Figure 1). The 7‐day risk windows were chosen to reflect the average duration of a single exposure period. Subgroup analyses were performed for users of tetracyclines, penicillins, cephalosporins, quinolones, macrolides and sulphonamides (and other combinations).

### The case‐crossover design

**Figure 2:**
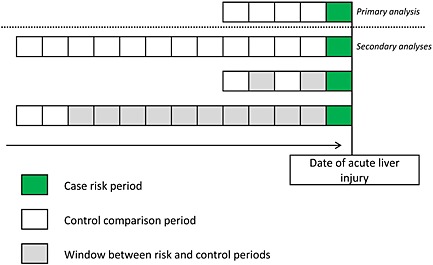
Diagram case‐crossover study ‘antibiotics and ALI’

The case‐crossover design shares similarities with a self‐controlled case series and case–control study design. As in the self‐controlled case series design, only patients that experienced the outcome of interest were considered, and each case acted as his or her own control. The cases, with exposed time before the onset of acute liver injury, and index dates in the case‐crossover analysis were the same as in our previous nested case–control design (see *Descriptive results*).^7^ The timing of risk periods in the case‐crossover design was dependent on the timing of the acute liver injury (like in a case–control study). Exposure to antibiotics was ascertained in five periods preceding the index date. Exposure in the case period, that is immediately prior to the acute liver injury, was compared to exposure in the past. The case period was defined as the 14 days immediately before the index date and this period was compared to four successive 14‐day cross‐over, or control periods starting immediately prior to the case period (see Figure 2). Again, subgroup analyses were performed by type of antibiotics.

### Secondary analyses

We conducted the following secondary analyses:

1. The risk and control periods that were used in the main self‐controlled case series and case‐crossover analyses were changed to make them more comparable with each other. For the self‐controlled case series analysis, the risk windows were changed to: (i) current use, (ii) a washout period of up to 90 days after the end of the last prescription and (iii) a washout period of up to 365 days after the end of the last prescription. We considered several risk periods for each patient in the case‐crossover study population. First, the case‐crossover case and cross‐over/control periods were extended to 30 days. Next, instead of four control periods, we used twelve 30‐day control periods. Last, we conducted a case‐crossover analysis with two rather than four control periods of 30 days each. We compared the impact of choosing two control periods immediately prior to the risk period versus two control periods a year before the risk period (see Figure 2).
2. To investigate a potential exposure time trend in the year before the event date, we conducted a control cross‐over analysis in which we included one control free of ALI for every case.
3. We conducted a SCCS analysis in which we removed the wash‐out periods to create more comparable observation periods.
4. A pre‐exposure period of 60 days was created to investigate short‐term event‐dependent fluctuations of exposure risk using the self‐controlled case series design.
5. There was a possibility that liver injury might increase the short‐term risk of death thereby leading to bias of an unpredictable nature. To examine the possibility of non‐random censoring of the follow‐up time we used a recently developed extension of the self‐controlled case series.^8^

### Statistical analyses

For the self‐controlled case series study relative incidence ratios were calculated by comparing the rate of acute liver injury experienced during risk periods with the rate of events during baseline time. Incidence rate ratios for the self‐controlled case series were calculated using conditional Poisson regression.^1^ The incidence rate ratio was adjusted for age in 1‐year agebands as age was considered a potentially important time‐varying confounder.

For the case‐crossover analysis we calculated Odds Ratios (OR) using conditional logistic regression by comparing exposure during risk windows (case person periods) with exposure during control periods.^3^

## Results Self‐Controlled Case Series Study

### Descriptive results

We identified 275 definite cases of acute liver injury for the self‐controlled case series. Seventy‐six cases were prescribed a single course of antibiotic agents during their follow‐up. The median duration of follow‐up was 6 years. A single exposure period lasted, on average, 7 days. The median age of patients at the time of their liver injury was 60.3 years, and 47% of all patients were male (see Table 1).

**Table 1:** Demographic details of the self‐controlled case series study population at the time of the recording of an incident acute liver injury

	First exposure					
Patient group (*n* = 275)	Before ALI	After ALI	Median duration of eligible follow‐up in years (95% CI)	Median age at first exposure in years (95% CI)	Median duration of exposure in days (95% CI)	Median age at time of ALI in years (95% CI)
All antibiotics	229	46	6.0 (5.64–6.0)	59.11 (56.0–61.87)	7 (7–7)	60.33 (56.46–63.32)
Males	104	26	5.9 (5.46–6.0)	59.10 (55.93–64.5)	7 (7–7)	60.48 (57.7–64.4)
Females	125	20	6.0 (5.59–6.0)	59.11 (52.85–63.78)	7 (7–7)	59.99 (53.76–64.9)

The same cases were included in the case crossover analysis, but 40 cases were prescribed antibiotics only after the date of acute liver injury. As the case‐crossover analysis relies on information from discordant pairs, these cases were omitted as they did not contribute any information to the analysis (not exposed during the case period and not exposed during the control period).

### Results self‐controlled case series study

The crude and age‐adjusted rate ratios that were found by using the self‐controlled case series design showed strong evidence of an increase in risk associated with the use of antibiotic agents. The rate ratio was highest during the first 7 days after receipt of a prescription (Rate Ratio [RR] 10.01, 95% CI 6.59–15.18) and remained increased the 7 days thereafter (IRR 5.18, 95% CI 1.61–16.66), see Table 2. There was strong evidence of an increased risk during the remaining exposed period and during the 30 days after the end of the last prescription (IRR 1^st^ 10 days after use: 5.67, 95% CI 3.61–8.89).

**Table 2:** Results self‐controlled case series analysis: the risk of definite ALI in users of antibiotics

	Patient population self controlled case series			
Exposure	Nr of cases	Person years	Rate ratio, unadjusted for age (95%CI)	Age‐adjusted rate ratio (95% CI)
Non use (baseline time)	182	1206.6	Ref	Ref
Current (0–7 days)	28	22.1	9.95 (6.57–15.07)	10.01 (6.59–15.18)
Current (7–14 days)	3	5.3	5.2 (1.62–16.7)	5.18 (1.61–16.66)
Current (15–30 days)	3	5.5	6.04 (1.83–20.0)	6.01 (1.81–19.91)
Current (>30 days)	6	19.3	6.52 (2.14–19.83)	6.05 (1.94–18.86)
Post‐exposure period (1–10 days after exposure)	23	32.0	5.66 (3.61–8.86)	5.67 (3.61–8.89)
Post‐exposure period (10–20 days after exposure)	17	30.8	4.35 (2.61–7.25)	4.35 (2.61–7.25)
Post‐exposure period (20–30 days after exposure)	13	28.2	3.59 (2.03–6.37)	3.59 (2.02–6.37)

When the self‐controlled case series analysis was stratified to allow for the estimation of relative risks of acute liver injury by agents grouped in broad classes, there were not enough cases to estimate the risk in users of tetracyclines and macrolides. Among the other agents the highest risk was found in users of cephalosporins (IRR 1^st^ 7 days after use: 19.78, 95% CI 9.31–42.03), followed by sulphonamides (IRR 17.79, 95% CI 8.15–38.82), penicillins (IRR 8.06, 95% CI 4.68–13.90) and quinolones (IRR 4.47, 95% CI 0.59–33.67—data not shown).

### Results case‐crossover study

The results of the case‐crossover analysis in which both risk and control windows were set at 14 days showed strong evidence of an increase in risk of acute liver injury during the 14 days before the index date as compared to the control periods up to 70 days prior to the index date (OR 3.05, 95% CI 2.06–4.53, Table 3).

**Table 3:** Results case‐crossover analysis: the risk of definite ALI in users of antibiotics

Exposure classification	Risk periods	Control comparison periods	Odds ratio	95% confidence interval
All antibiotic agents	Four control periods			
*Primary analysis:*				
Unexposed (ref)—*14 days*	47	324	1	Ref
Exposed	67	132	3.05	2.06–4.53
*Secondary analyses:*				
Unexposed (ref)—*30 days*	44	390	1	Ref
Exposed	84	119	4.82	3.30–6.05
	11 control periods			
Unexposed (ref)—*30 days*	92	1720	1	Ref
Exposed	88	338	5.17	3.76–7.09
	Two control periods with two windows of a month in between:			
Unexposed (ref)—*30 days*	26	173	1	Ref
Exposed	81	41	5.44	3.48–8.5
	Two control windows 1 year before the risk period:			
Unexposed (ref)—*30 days*	20	30	1	Ref
Exposed	75	154	6.5	3.95–10.71

When exposure was stratified by antibiotic class, the highest risk was found for quinolones (OR 6.92, 95% CI 1.29–36.96) and cephalosporins (OR 6.8, 95% CI 2.53–18.24), followed by tetracyclines (OR 3.27, 95% CI 0.68–15.68), sulphonamides (OR 3.19, 95% CI 1.42–7.17) and penicillins (OR 2.58, 95% CI 1.58–4.22—data not shown). Using the case‐crossover approach, the risk of definite ALI in users of macrolides was non‐significant (OR 1.34, 95% CI 0.42–4.24).

### Results secondary analyses

1. When the risk periods of the self‐controlled case series analysis were collapsed to allow for the measurement of current use of antibiotics, followed by ‘washout periods’, the main results did not change: The IRR of acute liver injury during current use was 11.6 (95% CI 7.57–17.79); the risk up to 90 days after the end of the last prescription was 4.02 (95% CI 2.92–5.55); and the risk from 90 to 365 days after the end of the last prescription was 1.86 (95% CI 1.33–2.59).
  When the risk and four control periods of the case‐crossover analysis were changed to 30 days, qualitatively similar results were found (OR 4.76, 95% CI 3.27–6.95). Slightly higher estimates were found when exposure to antibiotics in the 30‐day risk period was compared with exposure in 12 previous consecutive 30‐day control periods: OR 5.17, 95% CI 3.76–7.09. The highest relative risk estimate was found when only two control periods of 30 days each 1 year prior to the risk period were retained in the analysis (OR 6.5, 95% CI 3.95–10.71), see Table 3.
2. Using a control cross‐over analysis, we found no evidence of an exposure time trend (OR 0.94, 95% CI 0.54–1.63).
3. Omitting the washout periods from the follow‐up time lowered the point estimate of risk during the 1^st^ 7 days of antibiotic use (IRR 7.2, 95% CI 4.78–10.84) and the 7 days thereafter (IRR 3.54, 95% CI 1.10–11.38).
4. There was no evidence that patients were at an increased risk of ALI during the 60 days before any new exposure period. The risk of acute liver injury during the pre‐exposure period was 1.07 (95% CI 0.52–2.20, *n* = 8). The addition of a pre‐exposure period did not alter the results of the main analysis.
5. The observation period of nine percent of all patients ended within 60 days of being diagnosed with acute liver injury. We found no evidence of bias because of non‐random censoring of the observation period (IRR 1^st^ 7 days of antibiotic use: 9.41, 95% CI 6.23–14.23 and IRR 1^st^ 10 days after use: 5.38, 95% CI 3.44–8.40).

## Discussion

The relationship between antibiotic agents and acute liver injury was investigated using two different case‐only study designs. The results of the self‐controlled case series and case‐crossover analyses showed strong evidence of an increase in the risk of liver injury during the use of antibiotics. This already established drug‐outcome association was mainly chosen to facilitate a comparison of two different case‐only designs. Using the self‐controlled case series approach, the rate ratio was highest during the first 7 days after receipt of a prescription (IRR 10.01, 95% CI 6.59–15.18). The relative risk remained very high (IRR = 4) during the period lasting to 90 days post‐exposure and the risk was still high (RR = 1.86) during the remainder of the year post‐exposure. Relative risk estimates in the case‐crossover analysis were consistently lower. The lowest estimate was found in the primary case‐crossover analysis when antibiotic exposure in the 14‐day risk period was compared to exposure in four consecutive 14‐day control periods immediately prior to the risk period (OR = 3.05, 95% CI 2.06–4.53). The highest estimate was found when two 30‐day control periods 1 year prior to the 30‐day ALI risk period were retained in the analysis: OR 6.5 (95% CI 3.95–10.71).

### Comparison of case‐only designs

In the primary analysis of the SCCS study a post‐exposure period of 30 days was included to represent a gradual shift from full exposure to an entirely unexposed state. The results of the secondary analyses showed that omission of these post‐exposure periods, by adding them to the baseline comparison time, somewhat narrowed the difference between the results of the SCCS and the primary case‐crossover analysis. Omitting the washout periods lowered the IRR to 7.2, which was similar to the OR found using the case‐crossover approach with control periods that were 11 to 13 months removed from the date of acute liver injury (OR = 6.5). The relatively low risk estimate that was found in the primary case‐crossover analysis (OR = 3.05), compared to the risk estimates found in the secondary analyses, confirms the increased risk of ALI during post‐exposure periods. The relatively low risk is reflective of the choice of a limited number of small control periods immediately prior to the risk period. When longer and more distant control periods were included in the case‐crossover analysis the odds ratios increased, thereby reducing the difference in estimates between the case‐only designs. There was strong evidence of an increased risk of ALI during the 30 days after the last day of recorded antibiotic use and even up until 365 days after the end of exposure. It is worth noting that if the post‐exposure risk of ALI is true, and by conservatively using the lower confidence interval of the SCCS analysis, the relative risk of ALI is increased 33% up to 1 year after antibiotic exposure. This would cause more than twice as many acute liver injuries as a true IRR of 11.6 during actual exposure.

We found one further study using the self‐controlled case series method to investigate potential associations between antibacterial agents and acute liver injury. Ferrajolo *et al.* investigated signals of potential drug induced liver injury and found a high risk in adolescents and children in periods of antibiotic use compared to non‐exposed periods (IRR ranging from 2.6 (95% CI 0.8–9.3) for use of cefaclor to 16.7 (95% CI 9.9–28.1) for phenoxymethylpenicillin).^9^

### Strengths and limitations

The results of the case‐only studies were compared with the adjusted results of a previously conducted case–control study and a cohort study.^7^ The risk periods and study populations were not directly comparable between study designs as the case‐only designs were used to analyse a temporal change in risk within persons rather than a difference in risk between persons.^10^ Still, the relative risk estimates using the case‐only and traditional designs were similar in direction: The IRR found in the self‐controlled case series was within the CIs for the cohort analysis (IRR 7.31, 95% 4.91–10.89), and the results of the case‐crossover analysis using 30‐day windows were within the CI of the adjusted case–control study (OR 5.70, 95% CI 3.46–9.36). This suggests that between person‐confounding was adequately accounted for in the case–control and cohort analyses. The case‐crossover and self‐controlled case series analyses both provided some advantages over the more traditional designs (cohort‐ and case–control study). Because comparisons were made within persons rather than between persons, non time‐varying confounding variables did not affect the results as these were implicitly controlled for.

Case‐only study designs are better placed to assess the effect of accurately dated treatments that are transient in nature. As all users in the case‐only designs used antibiotics intermittently, with most cases (*n* = 120) having received two to four prescriptions over a six‐year period, antibiotics were an ideal drug to perform a case‐crossover and a self‐controlled case series design. A specific assumption of the case‐crossover design that needs to be met is that the exposure distribution in successive time periods should be exchangeable. We did not identify an exposure time trend nor any evidence of seasonality of exposure. Indeed, when we tabulated first exposure to antibiotics per month, the treatment was distributed roughly equally over all months, with slightly more users in April, May and December (11%) versus all other months (7%). We were not able to detect patients who were prescribed antibiotic agents outside primary care and we were not able to confirm when and whether a patient actually took the drug.

A specific assumption of the self‐controlled case series method is that an event should not affect exposure. If cases had been less likely to receive antibiotic agents for a short period after acute liver injury, then the time included after the liver injury would have been skewed towards non‐exposed time thereby introducing bias. This would tend to lead to an overestimate of the relative risk during the exposed period. The case‐crossover study would clearly not be affected by this bias as follow‐up time is censored at the time of the event. When we accounted for a potential change in prescribing patterns after a diagnosis of ALI by creating a pre‐exposure period, the relative risk in the pre‐exposure period was close to 1.00, suggesting no evidence of a short‐term change in the probability of antibiotic use after ALI.

We acknowledge that the results of both case‐only designs may have been confounded by a temporal change in underlying health at the time of their receipt of an antibiotic prescription. A limitation of our studies was the uncertainty with regard to the timing of the outcome as well as the validity of the outcome. The time of onset of acute liver injury was the date that a case presented with symptoms. A delay in the recording of hospital events in primary care records or a delay in patients presenting symptoms to their general practitioner could have affected the risk estimates. Indirect evidence for this theory can be seen with the continued increased relative risk observed in the self‐controlled case series at least 90 days after antibiotic discontinuation (4.02, 95% CI 2.92–5.55). By contrast, in the case crossover analysis, a delay in liver injury recording would tend to reduce the proportion of case periods containing an antibiotic exposure, whilst earlier control periods would be more likely to contain an antibiotic exposure. The result of this would be to bias the odds ratio towards the null; in our analysis the case‐crossover analysis yielded the lowest estimate of relative risk, suggesting the timing of event records may well have affected our results.

Whilst case‐only designs may not entirely be suitable to study transient acute events for which an accurate date of onset cannot be determined using clinical records, we believe that cohort and case–control designs would be prone to bias because of fixed confounders, whilst still being affected by the inaccurate timing of the event of interest.

## Conclusion

An increased relative risk of acute liver injury was consistently observed using both self‐controlled case series and case‐crossover designs. Both designs eliminate between‐person confounding, but other biases may possibly have affected the results. Case‐only designs can be used as a viable alternative study design to study the risk of acute liver injury, albeit with some limitations.

## Conflict of Interest

The authors declared no conflict of interest.

Key Points

- The results of the self‐controlled case series and case‐crossover showed strong evidence of an increase in the risk of acute liver injury during the use of antibiotics *and* several months post‐exposure.
- The age‐adjusted risk ratio estimates found in the self‐controlled case series study during the first days during and after antibiotic use suggested a stronger association than the results of the case‐crossover study: When longer and more distant control periods were included in the case‐crossover analysis the difference in estimates between the case‐only designs was reduced.
- The results suggest that both designs can be very informative, but biases can arise when assumptions are not met. Comparability of the designs depends on the choice, timing and duration of risk and control periods.

## Ethic Statement

The LSHTM ethics committee approved the study in May 2011 (5973). CPRD ISAC approval for the study was obtained in October 2011 (11_019A_2).

## Author Contributions

All authors contributed to the study conception and design. RB performed the data extraction and raw data analysis for the case only design studies. RB wrote the first draft and all authors contributed with critical comments to the final version.
